# A chromosome-scale and haplotype-resolved genome assembly of tetraploid blackberry (*Rubus* L. subgenus *Rubus* Watson)

**DOI:** 10.1093/hr/uhaf052

**Published:** 2025-02-18

**Authors:** Dev Paudel, S Brooks Parrish, Ze Peng, Saroj Parajuli, Zhanao Deng

**Affiliations:** Gulf Coast Research and Education Center, Department of Environmental Horticulture, University of Florida, IFAS, 14625 County Road 672, Wimauma, FL 33598, USA; Gulf Coast Research and Education Center, Department of Environmental Horticulture, University of Florida, IFAS, 14625 County Road 672, Wimauma, FL 33598, USA; Gulf Coast Research and Education Center, Department of Environmental Horticulture, University of Florida, IFAS, 14625 County Road 672, Wimauma, FL 33598, USA; Gulf Coast Research and Education Center, Department of Environmental Horticulture, University of Florida, IFAS, 14625 County Road 672, Wimauma, FL 33598, USA; Gulf Coast Research and Education Center, Department of Environmental Horticulture, University of Florida, IFAS, 14625 County Road 672, Wimauma, FL 33598, USA

## Abstract

Blackberries (*Rubus* spp.) are globally consumed and well known for their rich anthocyanin and antioxidant content and distinct flavors. Improving blackberries has been challenging due to genetic complexity of traits and limited genomic resources. The blackberry genome has been particularly challenging to assemble due to its polyploid nature. Here, we present the first chromosome-scale and haplotype-phased assembly for the primocane-fruiting, thornless tetraploid blackberry selection BL1 (*Rubus* L. subgenus *Rubus* Watson). The genome assembly was generated using Oxford Nanopore Technology and Hi-C scaffolding, resulting in a 919 Mb genome distributed across 27 pseudochromosomes, with an N50 of 35.73 Mb. This assembly covers >92% of the genome length and contains over 98% of complete BUSCOs. Approximately, 58% of the assembly consists of repetitive sequences, with long terminal repeats being the most abundant class. A total of 87,968 protein-coding genes were predicted, of which, 82% were functionally annotated. Genome mining and RNA-Seq analyses identified possible candidate genes and transcription factors related to thornlessness and the key structural genes and transcription factors for anthocyanin biosynthesis. Activator genes including *PAP1* and *TTG1* and repressor genes such as *ANL2* and *MYBPA1* play an important role in the fine tuning of anthocyanin production during blackberry development. Resequencing of seven tetraploid blackberry cultivars/selections with different horticultural characteristics revealed candidate genes that could impact fruiting habit and disease resistance/susceptibility. This tetraploid reference genome should provide a valuable resource for accelerating genetic analysis of blackberries and facilitating the development of new improved cultivars with enhanced horticultural and nutritional traits.

## Introduction

Blackberries (*Rubus* spp.) belong to the genus *Rubus* subgenus *Rubus* (formerly subgenus *Eubatus*) within the Rosaceae family [1]. They are characterized by their dark purple to deep black color, compound fruit structure, and a combination of juicy, tart, and sweet flavors. Blackberry fruits are an exceptional source of anthocyanins, antioxidants, and dietary fibers, offering significant health benefits to consumers. Over the past two decades, a sharp increase in consumer demand has led to substantial expansion of the market for fresh and processed blackberries in the USA and other countries worldwide [2]. As the fourth most economically important berry crop in the USA, the country produced 16 850 metric tons (MT) of processed and 1360 MT of fresh blackberries in 2017 [3]. In 2021, the USA imported 122 873 MT of fresh blackberries and 16 738 MT of frozen blackberries, valued at $519 million and $43 million, respectively. The global production of blackberries is estimated to be over 900 000 MT, making it a substantial contributor to the international berry industry. The ongoing development and introduction of new, improved cultivars has been instrumental in addressing consumer demands and increasing blackberry production across the globe.

Blackberries possess several interesting biological characteristics that distinguish them from other plants, including biennial growth habit, thorny canes, compound fruit structure, wide adaptation, and high hybridization potential with other species in the *Rubus* genus, which make blackberry species very interesting for studying the biology of these characteristics. Blackberry species have a significant amount of genetic diversity, which is exhibited by varying ploidy levels and chromosome numbers, ranging from diploids (2*n* = 2*x* = 14) to dodecaploids (2*n* = 12*x* = 84) [4]. Notably, many blackberry cultivars are tetraploids [4–6] and tetraploid cultivars form the foundation of major breeding programs and numerous genetic studies [7–9].

The primary goal of blackberry breeding is to develop high-yielding, thornless cultivars that produce berries with superior qualities and sweet flavors [1, 2, 10]. Shoots or canes of blackberries are covered from base to tip with thorny protrusions. Thorns on blackberry leaves and shoots cause damage to berries, increase disease incidences, and reduce marketable yield. Thorns also make it challenging to harvest berries, prune plants, and present hazards to farm workers. Thorns that contaminate machine-harvested fruit can be extremely difficult to remove and can make the product not only unappealing but also dangerous [11]. Thornlessness is essential for new blackberry cultivars to be widely adopted and grown by growers. Consequently, understanding the genetic control of blackberry thorns has been an important area of study in blackberry genetics.

A major breakthrough in blackberry breeding in recent years is the development and release of primocane-fruiting cultivars. While the majority of blackberries bear fruit on second-year canes, known as floricanes, primocane-fruiting blackberries can produce fruit on first-year canes. This trait offers remarkable advantages, including the potential for an extended harvest season, increased yield, and the ability to grow in regions with shorter growing seasons or colder climates [1]. Consequently, this character has the potential to revolutionize blackberry cultivation, significantly benefiting the global berry industry and consumers. The primocane fruiting trait was first discovered in the 1940s in wild blackberry [1]. Decades of breeding and selection efforts led to the release of the first commercial primocane-fruiting blackberry cultivar, ‘Prime-Ark® 45’, in 2009 [12] and a model for inheritance of primocane fruiting has been proposed [13]. A more comprehensive understanding of the genetic control and expression of this game-changing trait along with associated flowering intensity and flowering time traits is much needed for blackberry breeding. The development of molecular markers would greatly assist in incorporating this trait with strong flowering intensity and early flowering into a wider range of cultivars, further expanding the benefits of primocane-fruiting blackberries.

In commercial cultivation, blackberries are susceptible to several diseases such as orange felt [14], anthracnose, cane blight, and leaf spot that require regular pesticide applications to maintain healthy plants and crops [15, 16]. Overreliance on chemical treatments not only increases production costs but also leads to negative environmental consequences, making the development of disease-resistant cultivars a high priority. Selection for disease resistance is still in its infancy in blackberries, and research into disease resistance genes (*R* genes) promises to significantly contribute to enhancing blackberry resistance to diseases. By identifying and selecting *R* genes, breeders can develop cultivars with inherent resistance to pathogens, reducing the need for pesticides and improving the sustainability of blackberry cultivation. This development is particularly important for the expansion of blackberry cultivation into subtropical regions, which presents new challenges due to increased disease pressures in these climates [2].

Despite their economic importance and health benefits, genomic resources for blackberries remain very limited, significantly impeding the utilization of molecular markers and genomic selection in breeding efforts. Within the *Rubus* genus, the genome sequences for the subgenus *Ideaobatus* include diploid black raspberry (*R. occidentalis*) ORUS 4114–3 [17–19], the red raspberry (*R. idaeus*) cultivar ‘Anitra’ [20], and the cultivars ‘Autumn Bliss’ and ‘Malling Jewel’ [21], as well as *R. chingii* [22]. For the subgenus *Rubus*, the genome of diploid *R. argutus* ‘Hillquist’ has recently been released [23]. The availability of this single diploid blackberry genome has enabled a successful genome-wide association study, leading to the identification of multiple quantitative loci (QTLs) and candidate genes for two highly important berry traits: fruit firmness and red duplet reversion (RDR; a postharvest disorder) [8]. However, publicly available genome sequence data for tetraploid blackberries of the subgenus *Rubus* are still lacking. Polyploid genomes, with multiple copies of each homologous chromosome, are inherently more complex and challenging to assemble and analyze than their diploid counterparts. Therefore, developing a high-quality genome assembly for tetraploid blackberries is essential to facilitate research and drive breeding advancements.

So far, all the published *Rubus* genome assemblies had merged homologous sequences into a single consensus, resulting in collapsed assemblies for each diploid species. While collapsed assemblies simplify genome representation, they lose allelic variations and structural differences among homologous chromosomes. With the increasing adoption of long-read sequencing and advanced scaffolding technologies, more genome assemblies are being separated, or resolved, into haplotypes. Such assemblies retain distinct sequences for each homolog, preserving the unique information of each allele. For tetraploid species like blackberries, a haplotype-resolved assembly—comprising four sets of assembled pseudochromosomes—is particularly valuable for plant breeding and genetic research. This type of assembly enables detailed analyses of allelic combinations and diversity, facilitates the identification of trait-associated genes and allele blocks, and enhances understanding of complex inheritance patterns. Given these benefits, developing haplotype-resolved or phased genome assemblies has become a top priority in other major crops in the Rosaceae family, including strawberries [24–26] and roses [27]. Haplotype-resolved genome assemblies in strawberry and rose have revealed critical insights into subgenome divergence, homologous exchanges, and selective sweeps—key factors in understanding polyploid genome evolution and domestication. These assemblies enable breeders to pinpoint trait-associated alleles with greater precision, accelerating the development of new cultivars with enhanced yield, disease resistance, and quality traits [24–27].

In this study, we present the first chromosome-length, haplotype-resolved genome assembly and annotation of tetraploid blackberry. We further re-sequenced the genomes of seven tetraploid blackberry cultivars and new selections with different horticultural characteristics and performed RNA-Seq analyses on cane, leaf, and berry tissues. Using these genome and transcriptome data, we investigated the genetic polymorphisms in blackberry cultivars (and selections) and identified candidate genes potentially associated with disease resistance/susceptibility, thornlessness, primocane fruiting, and anthocyanin production in blackberry. These findings, along with the reference genome, can serve as a critical resource for discovery and analysis of genetics behind important traits in blackberries, ultimately accelerating molecular breeding efforts in this important berry crop.

**Figure 1:**
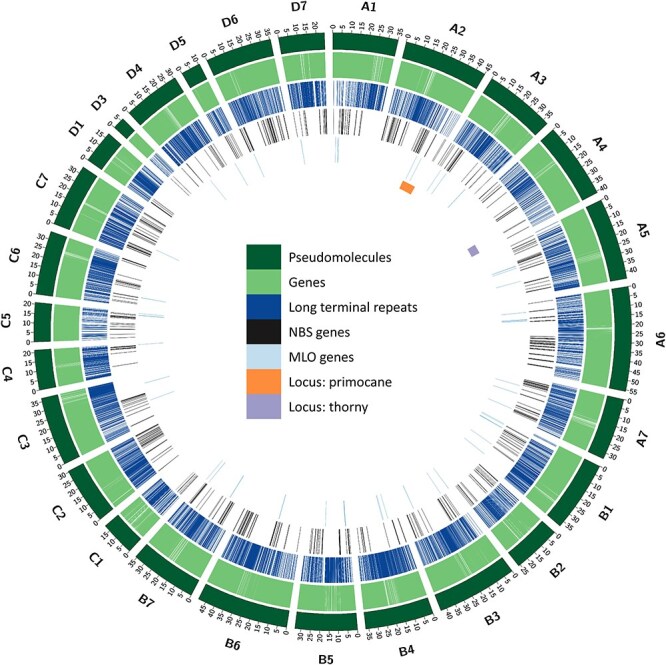
Circos plot showing the characteristics of the *Rubus* L. subgenus *Rubus* Watson BL1 genome. Concentric circles from outside to inside show the following: 1) 27 assembled pseudomolecules (in Mb); 2) Locations of predicted gene models; 3) Locations of predicted LTR TEs; 4) Locations of predicted nucleotide-binding site leucine-rich repeat class of disease resistance genes (NLR); 5) Locations of predicted disease susceptibility *MLO* genes; and 6) Locations of the loci genetically mapped for primocane fruiting (orange) and thornlessness

**Table 1:** Summary statistics of the BL1 genome assembly and annotation.

Total size of genome assembly	919.16 Mb
Chromosome number (2*n*)	28
Number of chromosomal pseudomolecules	27
Longest scaffold	55.81 Mb
Scaffold N50 length	35.73 Mb
Number of unscaffolded contigs	328
Size of unscaffolded contigs	42.36 Mb
Contig N50 length	241 kb
GC content	36.97%
Number of gene models	87 968
Number of genes with isoforms	6314
Mean transcript length	2179 bp
Mean coding sequence length	1084 bp
Mean exons per transcript	4.2
Number of transcripts	94 936
Repetitive elements	58.27%

## Results and discussion

### Genome sequencing, assembly, and annotation

A phased tetraploid reference genome for *Rubus* subgenus *Rubus* Watson BL1 selection (BL1) was *de novo* assembled using a combination of Oxford Nanopore sequencing (811× coverage of the monoploid genome, 160× coverage of the tetraploid genome) and Illumina sequencing (118× coverage of the monoploid genome) to produce highly contiguous pseudochromosomes (Fig. 1, Table 1). Utilizing the Hi-C chromosome conformation capture (Supplementary Fig. S1), 95% of contig sequences were organized onto 27 chromosomes for the four haplotypes. The total length of the final assembly was 919 165 722 bases distributed across 27 chromosome-level pseudomolecules (291 667 923; 262 227 137; 196 366 124; and 125 536 233 bases for haplotypes A, B, C, and D, respectively). The chromosomes were numbered sequentially based on their synteny to the diploid blackberry Hillquist genome [23], and the subgenomes were labelled A-D based on their length (Supplementary Fig. S2, Supplementary Fig. S3). The largest scaffold was 55.81 Mb (Chr A6), and the overall N50 was 35.73 Mb, corresponding to the median chromosome length (Table 1). The estimated GC content was 36.97%. Evaluation of the genome’s completeness using 2326 eukaryotic genes in the BUSCO OrthoDB10 eudicot dataset showed that the BL1 genome was 98.1% complete in gene space (Supplementary Table S2). Several chromosomal rearrangements are visible in the BL1 genome when compared to the Hillquist genome (Supplementary Fig. S2 and Supplementary Fig. S3). Overall, high collinearity was observed among the homologous chromosomes (Supplementary Fig. S4). Several translocation events were observed between the homologous chromosomes based on gene arrays (Supplementary Fig. S4). The assembled Chromosome D3 was short (~6.5 Mb) and was collinear with genes present in Chromosome C6 (Supplementary Fig. S4). Nevertheless, the majority of the DNA sequence in Chromosome D3 showed high synteny to the ends of the Ra03 chromosome in the Hillquist genome (Supplementary Fig. S2 and Supplementary Fig. S3). Telomeric repeats were identified towards one of the distal ends of 17 chromosomes, while telomeric repeats were identified at both ends of two chromosomes (A4 and B7) (Supplementary Fig. S5). In summary, the high completeness, large N50 length, and identification of telomeres indicate high quality of this genome assembly. However, further investigation is necessary to confirm or refute some of these rearrangements.

 Chromosome staining confirmed 28 chromosomes in the BL1 root tip somatic cells (Supplementary Fig. S6). Flow cytometry analysis of BL1 leaf cells revealed that its holoploid nuclear DNA content (2C) was 1.46 ± 0.05 pg, which corresponds to the monoploid 1C_x_ genome size of 0.365 pg (356.97 Mb) (Supplementary Fig. S7). This estimate is similar to the genome size of diploid *R. argutus* ‘Hillquist’ (337.4 Mb, 1C = 0.345 pg) [23]. The 2C nuclear DNA content of diploid *Rubus* species from the *Rubus* subgenus ranges from 0.59 pg (*R. hispidus* and *R. canadensis*) to 0.75 pg (*R. sanctus*) [6].

The *k*-mer analysis with GenomeScope 2.0 estimated the monoploid genome size of BL1 at 247 Mb, based on the tetraploid model. The calculated *aaab* value was greater than the *aabb* value*,* suggesting an autopolyploid nature for BL1 (Supplementary Fig. S8). This nature was corroborated by the smudgeplot with a bright smudge at AAAB (0.52) (Supplementary Fig. S9).

In blackberries, the first genetic linkage map was constructed using 119 simple sequence repeat (SSR) markers [7]. These markers were sufficient for constructing a framework of the blackberry genome. Recently, a maternal haplotype map consisting of 30 linkage groups was developed for blackberry using 2935 single nucleotide polymorphism (SNP) markers [23]. We mapped these SNP markers to the BL1 genome assembly. Most of the linkage groups displayed a one-to-one relationship with the pseudo chromosomes in the BL1 genome (Supplementary Fig. S10). However, several pseudochromosomes had fewer SNPs that were genetically mapped, which might be due to differences in the genome architecture or low numbers of SNPs in the linkage groups.

Currently, only the genome of one diploid blackberry ‘Hillquist’ has been published [23]. Compared to the Hillquist assembly (N50 = 38.6 Mb, and the maximum scaffold length = 45.5 Mb), this tetraploid genome assembly has a similar N50 of 35.75 Mb and a larger maximum length of 55.81 Mb (Chromosome A6) (Supplementary Table S1). We were able to incorporate 95% of the contig sequences into 27 chromosomes for the four haplotypes of the tetraploid blackberry, compared to seven collapsed chromosomes in the Hillquist genome. Based on the estimated genome size through *k*-mer analysis, the BL1 genome covers more than 92% of the genome length and contains over 98% of complete BUSCOs (Supplementary Table S2), which indicates a high level of completeness. In the BL1 genome assembly, repetitive elements account for 58.27% of the genome, slightly higher than the repeat content in the Hillquist genome (52.8%) (Supplementary Table S3).

Inherent polyploidy in tetraploid blackberries makes it challenging to assemble and accurately phase the genomes. While using Nanopore with Hi-C has enabled us to phase most of the genome (>90%), we were still unable to assemble one chromosome (Chr D2), which most likely collapsed during the assembly phases. This collapse is likely due to the chromosome being highly similar to the other homologous chromosomes, causing the assembler to treat the chromosome as if it were one of the others. This chromosome appears to be significant from the perspective of disease resistance, as it contains the highest number of nucleotide-binding sites and leucine-rich repeat (NLR) containing genes. Consequently, it is likely that this chromosome was highly conserved, leading to its collapse in the assembly. In addition, Chr D3 (6.5 Mb), Chr D5 (10.5 Mb), Chr D1 (17.9 Mb), and Chr C1 (18.6 Mb) were relatively shorter, thus likely incomplete and underrepresented in the current assembly.

### Genome annotation

The BL1 genome contains 58.27% repetitive elements (Supplementary Table S3). Long terminal repeat (LTR) transposable elements are the most predominant class (30.07%) while other repetitive elements are present in much smaller proportions, including DNA elements (1.88%), long interspersed nuclear elements (LINEs) (0.96%), and short interspersed transposable elements (SINEs) (0.01%). An integrated strategy combining *ab initio* and the homology-based methods was employed to predict gene models in the genome. We used RNA-Seq data from this study as well as from public databases and proteins from the Hillquist genome to make accurate gene predictions. In total, we identified 87 968 protein-coding genes with 94 936 transcripts (Table 1). The mean gene length is 2179 bp, and the mean coding sequence length is 1515 bp, with an average of 4.2 exons per transcript. The total gene length (206 900 785 bp) accounts for 22.5% of the BL1 genome. A total of 6314 genes have spliced isoforms, with an average of 2.1 isoforms per gene. The BL1 assembly had 87 968 total genes compared to 38 503 protein-coding genes in the Hillquist genome assembly. Functional annotation was assigned to 82% of the genes identified in the genome.

### Comparative genomics within the Rosaceae

**Figure 2:**
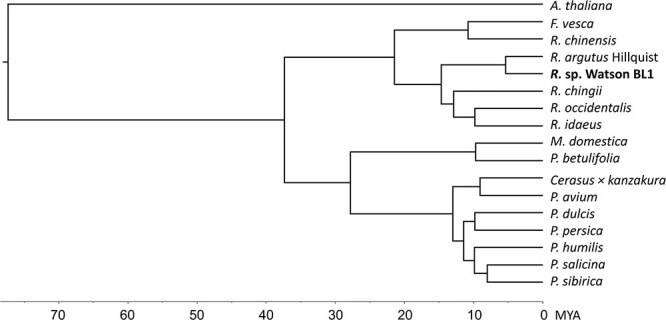
Phylogenetic tree of the BL1 blackberry and 15 plant species within the Rosaceae family inferred from published genome sequences. The genome of *Arabidopsis thaliana* was included as an outgroup. *X*-axis represents estimated divergence times (millions years ago, MYA) for the different lineages of Rosaceae plant species. *F*. *vesca* = *Fragaria vesca* (woodland strawberry, diploid) [28]; *R*. *chinensis* = *Rosa chinensis* (‘Old Blush’, doubled haploid) [29]; *R*. *argutus* Hillquist = *Rubus argutus* ‘Hillquist’ (blackberry, diploid) [23]; *R*. sp. Watson BL1 = *Rubus* subgenus *Rubus* Watson BL1 selection (tetraploid); *R*. *chingii* = *Rubus chingii* (Fepenzi variety ‘Wanfu 1’, diploid) [22]; *R*. *occidentalis* = *Rubus occidentalis* (black raspberry selection ORUS 4115–3, diploid) [18]; *R*. *idaeus* (red raspberry variety ‘Anitra’, diploid) [20]; *M*. *domestica* = *Malus domestica* Borkh. (apple variety ‘Golden Delicious’, doubled haploid) [30]; *P*. *betulifolia* = *Pyrus betulifolia* (Oriental pear, diploid) [31]; *Cerasus* × *kanzakura* (flowering cherry) [32]; *P*. *avium* = *Prunus avium* (sweet cherry, diploid) [33]; *P*. *dulcis* = (almond, diploid) [34]; *P*. *persica* = *Prunus persica* (peach cv ‘124 Pan’, diploid) [35]; *P*. *humilis* = *Prunus humilis* (variety ‘Jing ou No.2’, diploid) [36]; *P*. *salicina* (Japanese plum variety ‘Sanyueli’, diploid) [37]; and *P*. *sibirica* = *Prunus sibirica* (Siberian apricot) [38]

The gene family assignment using existing annotations for the 15 published Rosaceae genomes were explored to gain a global view of gene families among closely related species in Rosaceae (Supplementary Table S4). A total of 680 617 (94.4%) genes from BL1 and the other 15 Rosaceae genomes were assigned to 46 182 gene families. There were 7802 (16.89%) gene families shared by these genomes, implying their conservation. In the BL1 genome, 82 900 (94.23%) genes were assigned to orthogroups, and 2019 orthogroups were species specific, containing 6188 genes. These unique genes in BL1 were further annotated using Blast2go. The highest number of direct gene ontology (GO) count unique to BL1 was found in biological categories such as cotyledon development, seed germination, epidermal cell differentiation, maintenance of floral organs, post-embryonic development, and regulation of cellular process (Supplementary Fig. S11). BL1 had the highest number of one-to-one orthologues with the Hillquist genome (9484) and the lowest number of orthologues with flowering cherry (*Cerasus* × *kanzakura*) (730) (Supplementary Table S4). There are 17 345 genes in the Hillquist genome that have a one-to-many relationship with orthologues in BL1 (Supplementary Table S5). Using the protein sequences of single-copy orthologs, a high-confidence phylogenetic tree was constructed (Fig. 2).

**Figure 3:**
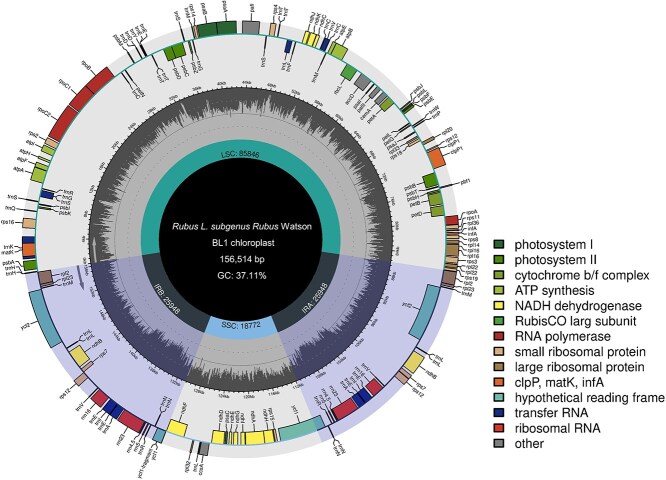
Chloroplast genome map of the BL1 blackberry with annotated genes. Genes inside the most outer circle are transcribed clockwise, and those outside the most outer circle are transcribed counterclockwise. Color coding of the genes is based on annotated functional groups. Boundaries of the SSC and LSC regions and inverted repeat (Ira and Irb) regions are denoted in the most inner circle

Within the four sub-genomes of BL1, a total of 74 366 (90.6%) of the genes were assigned to 20 423 orthogroups (Supplementary Table S6). Sub-genome A shared the highest number of one-to-one orthologues with Sub-genome B (8650 genes) (Supplementary Table S7).

### Chloroplast genome

The chloroplast genome of BL1 was assembled into a single contig of 156 514 bp with a GC content of 37.11% (Fig. 3). The chloroplast genome contains 160 genes, including 97 protein-encoding genes, 10 rRNA genes, and 65 tRNA genes. The chloroplast genome displayed a typical quadripartite structure, with one large single copy (LSC) region (85 846 bp) and one small single copy (SSC) region (18 772 bp) separated by two inverted repeat (IR) regions (25 948 bp each). The BL1 chloroplast genome was in high concurrence with the published chloroplast genome of a hybrid blackberry ‘Arapaho’, which was 156 621 bp with 134 genes [39]. For the various sequenced blackberry cultivars/lines in this research, the plastome lengths ranged from 156 514 to 156 704 bp with similar GC contents (36.4%–37.4%). These chloroplast genome sizes are comparable to the chloroplast genomes of other *Rubus* sp. including *Rubus peltatus* (155 582 bp) [40], *Rubus setchuenensis* (156 231 bp) [41], and *Rubus lambertianus* (156 569 bp) [42].

**Figure 4:**
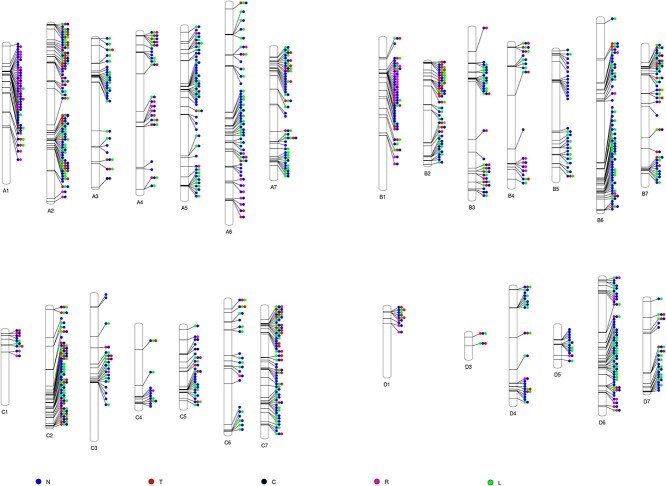
Distribution of NLR genes in the BL1 genome. Each vertical bar represents an assembled pseudochromosome in the BL1 genome. Blue dots represent genes with the NB-ARC domains, red dots the TIR domains, black dots the CC domains, green dots the RPW8 domains, and lime dots the LRR domains

**Figure 5:**
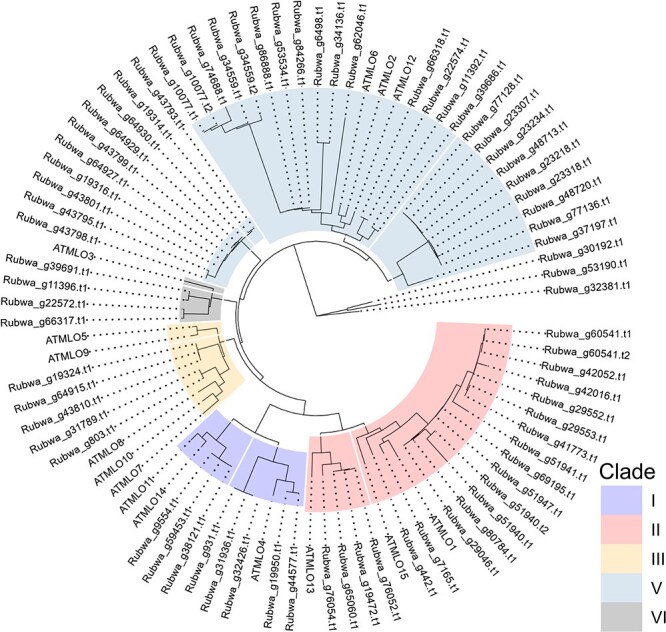
Clustering and phylogeny of *MLO* genes in the BL1 genome (prefixed with Rubwa) and *MLO* genes in *Arabidopsis thaliana* (prefixed with ATMLO)

### Disease resistance and susceptibility genes

Blackberry cultivation, particularly in the subtropical regions, faces major challenges from disease pressure [43, 44]. These diseases include leaf rust caused by *Kuehneola uredinis* [45], leaf spot caused by *Pseudocercospora pancratii* [44], Fusarium wilt caused by *Fusarium oxysporum* [46], downy mildew caused by *Peronospora sparsa* [47], powdery mildew caused by *Podosphaera aphanis* [48, 49], and orange cane blotch caused by the algae *Cephaleuros virescens* [14]*.* Developing disease-resistant varieties is crucial for helping growers address diseases in a socioeconomically and environmentally sustainable manner. Therefore, identifying disease-resistance genes is essential for facilitating breeding for disease resistance in blackberries [50]. The NLR class of *R* genes in plants is characterized by nucleotide-binding site (NBS) and leucine-rich repeat (LRR) domains, as well as variable amino- and carboxy-terminal domains [51, 52]. Analyzing NLR genes is crucial as they represent the largest class of *R* genes and play a significant role in plant resistance to multiple diseases caused by bacterial, fungal, and viral pathogens. In addition, we investigated the *Mildew resistance Locus O* (*MLO*) genes, a type of disease susceptibility (*S*) genes whose mutation has been found to confer broad-spectrum resistance against powdery mildew [53]. Studying *MLO* genes is important because they offer an opportunity to edit *S* genes and develop varieties with improved resistance to powdery mildew, an emerging disease in Serbia [49] and Mexico [48], two important blackberry-producing countries.

A total of 856 transcripts (770 genes) were identified as NLR genes in the BL1 genome, with the highest number of NLR genes found in chromosome A2 (60, 7.7%), followed by B6 (57, 7.4%) (Fig. 4). These NLR genes were further classified into different classes based on the presence of NB-ARC, Toll/Interleukin-1 receptor (TIR), coiled coil (CC), RPW8, and LRR domains (Fig. 4). The largest class, with at least one NB-ARC domain and one of the other domains was NL (265, 34.41%), followed by TNL (160, 20.77%) (Supplementary Fig. S12). The smallest class was NC, which contained the NB-ARC domain and the coiled coil (CC) domain (5, 0.64%). Most of these genes were clustered together (Fig. 4), and all the chromosomes contained at least two NLR genes. Chromosomes A2 and B6 contained the highest number (60 and 57) of NLR genes, indicating that these chromosomes are crucial for disease-resistance-related traits. The frequency of NLR domains was similar to that of the Hillquist genome, which contains a total of 228 genes with NLR domains, with NL and TNL as the largest classes (Supplementary Fig. S13). The highest number of complete NLRs in the Hillquist genome was found in Ra02 (46, 20.17%), followed by Ra05 (40, 17.54%), Ra06 (33, 14.47%), and Ra07 (33, 14.47%) (Supplementary Fig. S13).

A total of 74 genes were identified as members of the *MLO* family (Fig. 1, Supplementary Fig. S14). Twenty-one chromosomes each contain at least one *MLO* gene, with the highest number of *MLO* genes in Chromosome B1 and B5 (7 each). Homologs of all *AtMLO* genes, except *ATMLO7*, *ATMLO9*, *ATMLO10, ATMLO14*, and *ATMLO15*, were present in the BL1 genome, with *ATMLO6* having the highest number of homologous genes (19 in 11 chromosomes and 2 in contigs). *MLO* genes form a relatively small family in blackberries compared to the NLR class of genes. Clade IV and clade V are known to be involved in susceptibility to powdery mildew in monocots and dicots, respectively [54]. In the BL1 genome, we did not identify any homologs of clade IV (Fig. 5); however, 37 genes belonging to clade V were identified in the BL1 genome (Fig. 5, Supplementary Table S8). These genes could act as susceptibility factors in blackberries and are potential candidates for follow-up studies to investigate whether silencing, gene editing, or loss-of-function mutations in these genes could lead to powdery mildew resistance.

### Expression patterns of NLR and *DMR6* genes in blackberries

The BL1 genome assembly was utilized to understand the expression patterns of the NLR class of *R* genes and *S* gene in different blackberry organs. We generated transcriptome sequencing data from both thorny and thornless blackberry canes. In addition, blackberry transcriptome sequencing data were downloaded from four NCBI BioProjects (PRJNA680622, PRJNA701162, PRJNA744069, and PRJNA787794). In these BioProjects, transcriptome data were generated for blackberries in different developmental stages (green, red, and black; or unripe and ripe), for those treated with the plant growth regulator ABA, and for blackberry leaf tissues collected from plants fertilized with different types of nitrogen fertilizers.

A total of 608 NLR genes exhibited expression in at least one of these RNA samples evaluated (Supplementary Fig. S15, Supplementary Table S9). More NLR genes were expressed in blackberry leaves compared to fruit or shoot tissue. A higher number of genes were expressed in unripe berries compared to ripe berries. Similarly, more NLR genes were expressed in green and red colored blackberries as opposed to black colored blackberries.

A total of 4046 transcripts, including 28 NLR genes, were upregulated in black colored berries, while 8518 transcripts, including 129 NLR genes, were downregulated in black berries. In a separate experiment, 3106 transcripts with 10 NLR genes were upregulated in ripe berries compared to 3771 transcripts with 14 NLRs downregulated in ripe berries. A total of 1333 transcripts were commonly upregulated in both black and ripe berries. Among them, two transcripts were from NLR genes (Supplementary Table S10). Similarly, 2009 transcripts were commonly upregulated in both green and unripe berries, with 22 transcripts being NLR genes (Supplementary Table S10). The gene structure of these common transcripts revealed that most of the transcripts had a single exon (Supplementary Fig. S16).

Blackberries are highly susceptible to downy mildew (DM) caused by *Peronospora sparsa* [47]. The *Downy Mildew Resistance 6* (*DMR6*) gene is the *S* gene for DM in *Arabidopsis* [55]. This gene encodes 2OG-Fe(II) oxygenase [55], and its mutation confers resistance to DM and other pathogens in *Arabidopsis* [55, 56]. Knocking out *DMR6* has resulted in reduced susceptibility or resistance to downy mildew [57–59] and a number of other diseases [60–63] in several crops. We mined the BL1 genome and identified 392 transcripts that contained the 2OG-Fe(II) oxygenase superfamily (PF03171) domain, out of which 350 also contained the DIOX_N domain (PF14226). A total of six *DMR6* transcripts were significantly upregulated in thorny blackberry shoots (Supplementary Table S11), while 26 *DMR6* transcripts were upregulated in black and ripe berries, and 14 *DMR6* transcripts were downregulated in black and ripe berries. The highest number of differentially expressed *DMR6* transcripts were present in Chromosome A2 (9 transcripts).

### Candidate genes in the thorniness locus region

Most blackberry species exhibit sparse to abundant thorny protrusions on canes. These thorns, which are modified lateral branches, grow from lateral buds and are derived entirely from the tissues outside the vascular cortex [1]. The crosstalk between auxin and cytokinin determines lateral bud activation, a precondition for thorn development [64]. Since domestication and commercial cultivation of blackberries, gardeners, horticulturalists, and growers have sought thornlessness to reduce injury risks during harvest and maintenance [65]. Thornless blackberries are also resistant to or much less affected by the blackberry rosette disease, also known as double blossom or witches’ broom [66]. However, thornlessness has been associated with undesirable characters such as cold susceptibility, semi-erect growth habit, late-fruiting, and partial sterility [9, 67].

A previous genetic mapping study identified one locus (*S* locus) for thorniness in blackberries [7]. This locus was mapped to a region of 8.8 Mb in chromosome A4 from 30 682 793 to 39 471 371 bp of the BL1 genome (Fig. 1). This genomic region contains 1567 genes, with a majority involved in processes such as response to chemicals (6.42%), response to stress (6.04%), anatomical structure development (5.37%), biosynthetic process (4.62%), and multicellular organism development (4.43%) (Supplementary Fig. S17). Among these genes, 49 genes have NLR domains, and two possess complete NLR domains (Rubwa_g15611.t1 and Rubwa_g15684.t1).

The thorniness locus region contains 255 transcripts with at least one HIGH impact variant (Supplementary Table S12). GO annotation of these transcript revealed a higher presence of those related to stress response, chemical response, anatomical structure development, biosynthetic process, and multicellular organism development (Supplementary Fig. S18). Ninety-six of the transcripts within the locus region contain transcription factors (Supplementary Table S13), including bHLH and MYB, which are involved in trichome development [68].

The bHLH family, a large group of transcription factors in *Arabidopsis*, include members such as *GLABROUS3* (*GL3*) and *ENHANCER OF GLABROUS3* (*EGL3*), which play a role in trichome development [69, 70]. MYB16- like genes (MIXTA-like R2R3-MYB family member) are involved in trichome initiation and regulate conical cell outgrowth in diverse plant species [71]. Chromosome 4 in red raspberry (*Rubus idaeus*) is also important for controlling prickle and shows differential expression of these transcription factors [72].

Several other important transcription factors, such as *AP2*, *DRF*, *MIKC*_*MADS*, *WRKY*, *NAC*, and *SBP*, were identified in the thorniness locus region of BL1 (Supplementary Fig. S19). In particular, NAC transcription factors have been implicated in spine development in cucumbers [73], while SBP genes control initiation of trichomes in rice [74].

Previously, we identified shoots (canes) on ‘Prime-Ark® 45’ (PA45) plants that expressed few short thorns and designated this variant as PA45m. We performed RNA-Seq analysis on PA45’s thorny canes and PA45m’s thornless canes and revealed 123 transcripts that were upregulated in thornless blackberries, with four of these transcripts found within the thorniness locus region (Supplementary Table S14). Meanwhile, 520 transcripts were downregulated in thornless blackberries, and 14 of these transcripts were located within the thorniness locus region. These transcripts have been functionally characterized as being involved in trichome development in other species. For instance, the pleiotropic drug resistance gene (*NpPDR1*) is constitutively expressed in the roots and leaf glandular trichomes of *Nicotiana plumbaginifolia*. These trichomes secrete terpenoid molecules as the first line of defense in response to plant pathogens [75]. Another differentially expressed gene, Caffeoyl-CoA, is involved in lignin biosynthesis in hops [76].

A downregulated transcript, Glycosylphosphatidylinositol-anchored lipid protein transfer 1 (Rubwa_g15399.t1), has been found to be expressed in the trichomes in *Arabidopsis* leaves, and its disruption leads to alterations in cuticular lipid composition [77]. In tomatoes, beta-ketoacyl reductase has been identified as trichome-enriched candidate gene [132].

The *DORNRÖSCHEN* (*DRN*) gene, also known as *ENHANCER OF SHOOT REGENERATION1* (*ESR1*) gene, contributes to the organization of meristems in *Arabidopsis* [78] and plays a role in cytokinin-independent shoot regeneration [79]. Interestingly, homocysteine S-methyltransferase 3, one of the top upregulated genes in the prickleless epidermis in *Solanum viarum* Dunal [80], was found to be downregulated in thornless blackberries.

We identified four transcripts significantly upregulated in thornless blackberries, shedding lights on their potential roles in thorn development. Lysine histidine transporters are involved in importing amino acids into trichomes and play a role in organic N transfer for pollen production [81]. MYB domain protein 16 was also upregulated in thornless blackberries. Interestingly, MYB16 was downregulated in prickle-free red raspberry [72]. The MIXTA-like transcription factor MYB16 is a major regulator of cuticle formation in vegetative organs [71]. Additionally, the MED28 mediator subunit is essential for both development and senescence processes [82]. Lastly, one of the transcripts of Glycosylphosphatidylinositol-anchored lipid protein transfer 1 (Rubwa_g15399.t2) was found to be upregulated.

These up- and down-regulated genes are potential candidate genes for regulating the thornlessness trait in blackberries. These findings may hold considerable importance from a breeding perspective. Information for these genes is provided in Supplementary Table S15. In prickle-free epidermis tissue of red raspberry, transcription factors such as MIXTA-like R2R3-MYB family members, MADS-box, AP2/ERF, and NAC were significantly downregulated [72]. We also identified these transcription factors in the thorniness locus region of blackberries (Supplementary Table S13, Supplementary Fig. S19), suggesting that the mechanisms for thorn initiation in these two species could be similar. Furthermore, in the thornless blackberries, we observed downregulation of two other NLR transcripts (Rubwa_g6165.t in chromosome A2, and Rubwa_g54129.t1 in chromosome C1) and upregulation of two NLR transcripts (Rubwa_g33903.t1 in chromosome B1, and Rubwa_g79199.t1 in chromosome D6).

### Candidate genes for primocane fruiting

The primocane fruiting trait was genetically mapped to one locus (*F* locus) between markers FF683693.1 Rh_MEa0007aG06 and FF683518.1 Rh_MEa0006aC04 in the blackberry genome map [7]. This region corresponds to an 11 Mb segment in the Hillquist chromosome Ra02 ranging from 25 901 374 to 25 901 083 bp (FF683518.1 Rh_MEa0006aC04) and 37 085 586 to 37 085 204 bp (FF683693.1 Rh_MEa0007aG06) [23]. In the BL1 tetraploid genome, Ra02 corresponds to Chromosome A2, and the region was mapped to a 12.5 Mb segment between 29 653 983 and 42 218 375 (Fig. 1). This region contains 2172 genes.

Comparison with the FLOR-ID database [83] revealed 32 flowering-related homologs in the BL1 genome within this locus region (Supplementary Table S16). All these genes were also identified in the Hillquist genome (Supplementary Table S16). These genes have been reported to have positive effects on flowering time, such as *FT*-*INTERACTING PROTEIN 1, IDD8*, *GA1*, *GID1B*, *LD*, *NF*-*YB2*, *SUS4*, *SKB1*, *FPF1*, *DCL3*, *MRG1*, and *GA20OX3*. Additionally, genes with negative effects on flowering time, such as *HTA*, *WDR5A*, *CSP2*, *TPL*, *UBP26*, and *GASA5*, were present in the locus region. A loss-of-function mutation in a repressor could result in recessive inheritance of this trait, making floral repressors the primary candidates for primocane fruiting [23]. As a result, these identified genes might play a critical role in primocane fruiting in blackberries and merit further investigation. We identified genes encoding transcription factors including C2H2 (Rubwa_g6736.t1), HB-other (Rubwa_g7150.t1), and NF-YB (Rubwa_g7154.t1) in the primocane-fruiting locus. Additionally, genes involved in the photoperiod pathway, including *FTIP1* (Rubwa_g6515.t1, Rubwa_g6515.t2, Rubwa_g6518.t1), *NF-YB* (Rubwa_g7154.t1), and *TPL* (Rubwa_g7556.t1, Rubwa_g7559.t1), were also identified within this locus region. Two *FAF* (FANTASTIC FOUR) genes were identified within the primocane fruiting loci (Rubwa_g6900, Rubwa_g6994). The *FAF* gene family has been recently connected with early flowering in tomato and is a promising candidate in blackberry [84].

### Candidate genes for anthocyanin biosynthesis

Anthocyanins are the key determinants of blackberry’s distinct color and strong antioxidant activities [85, 86]. We analyzed the anthocyanin biosynthesis pathway in tetraploid blackberry through RNA-Seq and compared the expression levels of anthocyonin biosynsthesis genes and regulator genes at three stages of fruit development: green, red, and black. The heat maps indicate variations in expression of key genes involved in the anthocyanin biosynthesis pathway across the three fruit developmental stages (Supplementary Fig. S20). The pathway starts with phenylalanine and progresses through several intermediates to produce anthocyanins and other flavonoids. In the early stages of fruit development (green stage), there is high expression of genes involved in the initial steps of the pathway, such as phenylalanine ammonia-lyase (*PAL*) and cinnamate-4-hydroxylase (*C4H*). Specifically, *PAL* genes (e.g. Rubwa_g22612, Rubwa_g35462) and *C4H* genes (e.g. Rubwa_g12045, Rubwa_g12494) show elevated expression in the green stage, which decreases as the fruit matures to red and black stages.

As the fruit progresses to the red stage, there is a noticeable shift in the expression of mid-pathway genes, including chalcone synthase (*CHS*) and chalcone isomerase (*CHI*). For instance, *CHS* genes (e.g. Rubwa_g12525, Rubwa_g12526) and *CHI* genes (e.g. Rubwa_g35644) exhibit high expression in the green and red stages but reduce in the black stage.

In the later stages (red to black), genes involved in the final steps of anthocyanin production, such as flavanone 3-hydroxylase (*F3’H*) and anthocyanidin synthase (*ANS*), showed increased expression. *F3’H* genes (e.g. Rubwa_g17314 and Rubwa_g62520) and *ANS* genes (e.g. Rubwa_g17912 and Rubwa_g3805) exhibit a marked increase in expression from the green to black stages, correlating with the accumulation of anthocyanins. Furthermore, UDP-glucose: flavonoid 3-o-glucosyltransferase (*UFGT*) gene (e.g. Rubwa_g30962) demonstrated significantly higher expression in the black stage, indicating its role in the final steps of anthocyanin biosynthesis and stabilization.

The transcription factors involved in anthocyanin biosynthesis show distinct expression patterns, indicating their roles as either activators or repressors of the pathway. Transcriptional activators, such as *PAP1*, and *TTG1*, display increased expression in the red and black stages. For instance, *PAP1* (e.g. Rubwa_g2120 and Rubwa_g33319) and *TTG1* (e.g. Rubwa_g71707, Rubwa_g7001) were more active in the red and black stages, suggesting their role in upregulating genes necessary for anthocyanin production during fruit ripening.

Conversely, certain transcription factors appear to function as repressors, showing higher expression in the green stage and decreasing as the fruit matures. For example, *ANL2* and *MYBPA1* showed higher expression in the green stage, which diminished in the red and black stages, indicating their potential role in repressing anthocyanin biosynthesis genes during early fruit development. These transcription factors (e.g. Rubwa_g68467, Rubwa_g30470, Rubwa_g51341, Rubwa_g51007, Rubwa_g80489) might help regulate the timing of anthocyanin production, ensuring it aligns with the later stages of fruit maturation.

Gene expression pattern from the early to the late stages of fruit development aligned with the visible accumulation of anthocyanins. Early-stage genes were downregulated as the fruit matured, while late-stage genes were upregulated, driving the production and accumulation of anthocyanin pigments in the blackberries. The distinct roles of transcriptional activators and repressors further refine the regulation of this biosynthetic pathway, ensuring precise control over anthocyanin production during fruit ripening. The identification of these important genes in the newly assembled genome will allow for further investigations into fruit quality.

### Sequence polymorphisms among tetraploid blackberry cultivars/selections

We resequenced the genomes of seven tetraploid blackberry cultivars/selections (‘Kiowa’, ‘Osage’, ‘Prime-Ark® 45’, PA45m (‘Prime-Ark® 45 variant with fewer and shorter thorns), ‘Prime-Ark® Freedom’, ‘Prime-Ark® Traveler’, and BL2 selection) to uncover sequence polymorphisms that may contribute to their diverse characteristics. We identified a total of 13 378 961 sequence variants, with an average rate of one variant every 65 bp (Supplementary Table S17). The majority of these variants were classified as SNPs (9 846 835, 73.59%), with insertions and deletions accounting for 9% (573 329 insertions and 641 516 deletions). Most SNP effects were classified as having a ‘MODIFIER’ putative impact (97.914%), while 36 553 SNP (0.072%) effects were considered to have a ‘HIGH’ putative impact, 464 161 SNP (0.909%) effects have a ‘LOW’ impact, and 564 543 (1.106%) effects have a ‘MODERATE’ putative impact. SnpEff also classified 463 095 (53.00%) as missense, 8004 (0.92%) as nonsense, and 402 724 (46.09%) effects as silent functional classes. Most of the variants were located in intergenic regions (21.428%) or upstream and downstream regions (32.191% and 31.452%, respectively), with 1.97% of the variants found in exons. Based on nucleotide substitutions, 20 524 771 SNPs were classified as transitions, and 11 448 387 SNPs were classified as transversions, resulting in a genome-wide transition to transversion ratio (Ts/Tv) of 1.79. A summary of the variants for individual blackberry samples is provided in Supplementary Table S17.

By comparing the variant information of leaf rust-susceptible cultivars ‘Kiowa’ and ‘Prime-Ark® Freedom’ to leaf rust-resistant cultivars ‘Osage’, ‘Prime-Ark® Traveler’, and ‘Prime-Ark® 45’, we identified 284 variants present in 54 NLR genes (Supplementary Table S18) that exhibited differences between leaf rust-resistant and susceptible cultivars. Notably, two SNPs were found in Rubwa_g15684.t1, located in the thorniness locus region. The analysis of these genetic differences may be crucial for understanding the mechanisms underlying disease resistance and thornlessness. It has been observed that thornless blackberries are much less susceptible or resistant to blackberry rosette (double blossom) disease [66]. It will be interesting to examine the association of these NLR genes at this locus with thornlessness.

The re-sequenced data from these seven blackberry cultivars/selections were also aligned to the Hillquist genome assembly to call sequence polymorphisms in tetraploid blackberries (Supplementary Table S19). We identified a total of 6 427 606 variants with the rate of 1 variant every 42 bases. Chr2 (Ra02) had the lowest variant rate (33) compared to the highest rate with Ra05 (49). The majority of these variants were classified as SNPs (4 037 029, 62.81%), with insertions and deletions accounting for 10.89% (372 338 insertions and 327 922 deletions). In comparison of the variants for the different genotypes based on the BL1 (Supplementary Table S17) or Hillquist genome (Supplementary Table S19) as the reference, we found that using the BL1 genome as the reference resulted in a greater number of SNPs (39.15% to 69.88% more) detected in most tetraploid blackberry genotypes as compared to using the Hillquist genome as the reference. On the contrary, SNP counts were lower in ‘Prime-Ark® Traveler’ (84.41%) and ‘Prime-Ark® Freedom’ (79.18%) when the BL1 genome was used as the reference for variant calling than when the Hillquist genome was used as the reference. This is expected because BL1 was a direct progeny of ‘Prime-Ark® Freedom’ and ‘Prime-Ark® Traveler’, and it shares a significant portion of its genome with both parents and there are fewer genetic differences between BL1 and its parents.

## Conclusion

In this study, we present the first chromosome-length, phased genome assembly and annotation of the tetraploid blackberry selection BL1. Comparisons with the published diploid Hillquist genome attest to the high quality of the BL1 genome, and BUSCO analysis further demonstrates its high completeness and comprehensiveness. Using comparative genomic approaches, we shed light on the relationship between BL1 and 15 other sequenced genomes within the Rosaceae family. We identified numerous disease resistance and susceptibility genes (NLR, *MLO*, and *DMR6*) in the BL1 genome, which will be instrumental for developing disease-resistant blackberry cultivars. Moreover, we discovered potential candidate genes and transcription factors associated with thornlessness and primocane-fruiting, which could aid in the development of new thornless, primocane-fruiting blackberry cultivars. Additionally, we revealed a clear progression in gene expression in the anthocyanin pathway from the early to the late stages of fruit development, aligning with the visible accumulation of anthocyanins. Resequencing seven blackberry cultivars/selections unveiled genetic diversity that can be harnessed for future breeding efforts. The BL1 genome sequence and annotation will serve as an invaluable resource for breeding, genetic, genomic, and molecular research in blackberries and related berry crops. The release of this tetraploid blackberry genome can contribute to more efficient and targeted breeding, ultimately leading to the development of new cultivars with enhanced fruit quality, desirable fruiting habits, as well as resistance to important diseases.

## Materials and methods

### Plant material and experimental design

The BL1 blackberry selection was chosen for genome sequencing and assembly because it resulted from a cross between ‘Prime-Ark® Traveler’ (PAT) [87] and ‘Prime-Ark® Freedom’ (PAF) [88]. ‘Prime-Ark® Freedom’ was the world’s first commercially released thornless primocane-fruiting blackberry, while ‘Prime-Ark® Traveler’ is the first thornless primocane-fruiting blackberry cultivar with good shipping quality. Both PAT and PAF were included in the genome resequencing. In addition, three other blackberry cultivars and two selections were re-sequenced: ‘Kiowa’ [89], ‘Osage’ [90], ‘Prime-Ark® 45 (PA45) [12], PA45m (variant of ‘Prime-Ark® 45′ with much fewer and shorter thorns; S. Parajuli and Z. Deng, unpublished), and the BL2 selection. These cultivars/selections represent substantial variations in thornlessness, fruiting habit, and resistance to leaf rust disease (Supplementary Table S20).

### Estimation of genome size

Young unexpanded leaves of BL1 blackberry were used for flow cytometry. The one-step protocol with Tris MgCl_2_ lysis buffer, RNase, and propidium iodide recommended by Doležel *et al.* (2007) [91] was followed for sample preparation. ‘Polanka’ soybean (*Glycine max* Merr.; 2.50 pg/2C) and ‘Stupické polní rané’ tomato (*Solanum lycopersicum* L.; 1.96 pg/2C) were used as internal standards. Three biological replicate samples were analyzed with three technical replicates per biological replicate. Holoploid DNA content (2C) was calculated as follows: DNA content of the standard x mean fluorescence value of blackberry sample / mean fluorescence of the internal standard. The monoploid genome sizes of blackberries were calculated by dividing the 2C genome size by the inferred ploidy level [92].

For chromosome squashing, root tips, approximately 1 cm, were isolated and treated in a 2 mM 8-hydroxyquinoline solution for 3 hours at 4°C. Pre-treated roots were washed with deionized water and placed in a fixative solution at 4°C overnight. The solution contained 3 methanol:1 glacial acetic acid (v/v). The next day, roots were washed with deionized water and macerated in 1 N HCl for 22 min. Acid was removed and root tips were washed a final time in deionized water. Macerated root tips were stained in 2% acetocarmine solution (Carolina Biology Supply Company) overnight. Root caps and non-meristematic tissue were removed under a stereoscope. Finally, root tips were squashed in a small drop of 2% acetocarmine solution with a cover slip on a glass slide. The slides were observed under a bright field microscope at ×1000 magnification.

### DNA extraction and genome sequencing

Young leaf tissues were collected from BL1 and several other blackberry genotypes and immediately preserved in liquid nitrogen. The frozen samples were transported on dry ice to NextOmics Biosciences (Wuhan, China) for DNA extraction and sequencing. High-molecular weight DNA was isolated using the Qiagen DNeasy® Plant kit. For Oxford Nanopore Technologies (ONT) sequencing, libraries were prepared using the SQK-LSK109 1D genomic DNA ligation sequencing kit according to ONT’s protocol (version GDE_9063_v109_revD_04Jun2018; Oxford Science Park, Oxford, UK) and sequenced on the PromethION platform. For Illumina sequencing, the TruSeq DNA PCR-free library preparation protocol (revision A, January 2013, with a 550 bp insert size) was used to construct PCR-free libraries, which were sequenced on a NovaSeq 4000. Supplementary Table S21 provides a summary of the sample details.

### RNA isolation, RNA-seq library preparation and sequencing

RNA extraction, mRNA enrichment, RNA-Seq library construction, and sequencing were performed by NextOmincs Biosciences and CD Genomics (Shirley, NY, USA). Total RNA was isolated using the Qiagen RNeasy kit (Qiagen, Switzerland) according to the manufacturer’s protocol. Two micrograms of RNA per sample were utilized for library construction, followed by sequencing on the Illumina NovaSeq4000 (NextOmics) or NextSeq platform (CD Genomics). RNA-Seq data were generated for shoots and leaves from BL1, as well as green berries from PA45, to support transcriptome profiling and genome annotation. Similarly, RNA-Seq was performed on triplicates of thorny shoot and thornless shoots from PA45m, and the data was utilized for differential gene-expression analysis and analysis of NLR genes. A summary of samples used in RNA-Seq is provided in Supplementary Table S21.

### Sequence data processing and genome assembly

The quality of ONT data was assessed using NanoPlot/1.30.1 (https://github.com/wdecoster/NanoPlot). Raw reads were trimmed to a –min_trim_size of 6 using porechop/0.2.4 (https://github.com/rrwick/Porechop). Several assemblers were evaluated for assembling these nanopore sequencing data, including canu/1.8 [93], wtdbg/2.5 [94], WENGAN/0.2 [95], SMARTdenovo/20180219 [96], NECAT/0.0.1 [97], and Flye/2.91.1 [98]. A summary of the assemblies is provided in Supplementary Table S22. Parameter optimization was performed for NECAT, and the assembly with the longest total length (setting3) and highest N50 (default) were chosen for Hi-C scaffolding (Supplementary Table S23). The final assembly was produced by Hi-C scaffolding on contigs of NECAT - setting3.

### Genome anchoring and scaffolding

Chromatin conformation capture data were generated using the Phase Genomics’ Proximo Hi-C 4.0 kit (Seattle, WA, USA), a commercial implementation of the Hi-C protocol. Blackberry leaf tissues were processed following the manufacturer’s instructions, beginning with formaldehyde crosslinking of intact cells to preserve chromatin interactions. The chromatin was digested with a combination of restriction enzymes (*Dpn*II, *Dde*I, *Hin*FI, and *Mse*I), end repaired with biotinylated nucleotides, and proximity ligated to form chimeric DNA molecules representing spatially adjacent genomic regions. Streptavidin beads were then used to isolate the biotin-tagged fragments, which were subsequently converted into Illumina-compatible sequencing libraries. Sequencing of the libraries on the Illumina NovaSeq platform yielded 78 642 356 paired-end reads (150 bp each).

Reads were aligned to the BL1 draft genome assembly following the manufacturer’s recommendations. Alignments were performed using BWA-MEM/0.7.17 [99] with the -5SP and -t 8 options enabled, while other parameters remained at their default settings. PCR duplicates were flagged using SAMBLASTER/0.1.24 [100] and subsequently excluded from downstream analysis. Filtering was conducted with samtools/1.15 [101] using the -F 2304 flag to remove non-primary and secondary alignments. Hi-C alignments were analyzed in Juicebox [102] to identify potential misjoins, leading to 101 breaks across 90 contigs. The revised assembly was subjected to the same alignment and filtering process to ensure consistency and accuracy.

Chromosome-scale scaffolds were generated from the corrected assembly using the Phase Genomics’ Proximo™ Hi-C genome scaffolding platform, following the principles outlined by Bickhart *et al.* [103]. Similar to the LACHESIS approach, this method constructed a contact frequency matrix from aligned Hi-C read pairs, normalized based on restriction site distribution on contigs. Scaffold construction was guided by optimizing expected contact frequencies and statistical patterns inherent in the Hi-C data. To refine scaffold accuracy and concordance with the Hi-C data, approximately 60 000 separate Proximo runs were conducted, iteratively improving scaffold structure and resolution.

### Annotation of repetitive elements

Repetitive elements (TEs) were annotated using a combination of tools. A *de novo* TE library was constructed with RepeatModeler/2.0 [104], while miniature inverted transposable elements (MITEs) were identified through MITE-Hunter/201111101 [105]. Long terminal repeat (LTR) retrotransposons were detected using LTR_finder/1.07 [106] and genometools/1.5.7 [107], with LTR_retriever/2.9 [108] to process the outputs. The predicted TEs were integrated into RepBase for annotation, and RepeatMasker/4.1.1 [104] was used for final TE annotations. The Telomere Identification toolkit (tidk) (https://github.com/tolkit/telomeric-identifier) was employed to identify telomeric repeat sequences, searching for the canonical repeat unit for the Rosales clade: AAACCCT.

### Gene prediction and annotation

Gene prediction was carried out using the BRAKER/v2 pipeline [109], utilizing the transcriptome generated from RNA-Seq of thorny shoots, thornless shoots, leaves, and green fruits in this study, and publicly available RNA-Seq data from PRJNA680622, PRJNA701162, PRJNA744069, and PRJNA787794. BRAKER was run with protein data from OrthoDB for Viridiplantae. The final gene predictions, integrating evidence from RNA-Seq and homologous proteins, were refined and extracted using TSEBRA [110].

### Chloroplast genome assembly and annotation

The chloroplast genome of BL1 was assembled using GetOrganelle v 1.7.5.0 [111] with Illumina short reads. Annotation was performed with GeSeq /2.03 [112], and a visualization of the annotated genome was generated using Chloroplot /0.2.4 [113].

### RNA-seq expression analysis of NLR genes

Raw RNA-Seq reads generated in this study (triplicates of thorny canes of ‘Prime-Ark® 45 and thornless shoots of PA45m) were processed using Trimmomatic/0.39 [114] to remove adapter and low-quality sequences. Public RNA-Seq datasets from PRJNA680622, PRJNA701162, PRJNA744069, and PRJNA787794 underwent the same trimming process. Read quality for all datasets was evaluated with FastQC/0.11.7 [115]. Clean reads were aligned to the BL1 genome assembly using STAR RNA-seq aligner version 2.7.9a [116], and transcript-level expression was quantified with StringTie/2.1.3b [117], which provided transcript per million (TPM) values. TPM values for NLR genes were extracted and visualized as a heatmap to compare expression levels across the samples.

### Differential gene expression of thorny and thornless shoots

Differential gene expression analysis was done by comparing thorny and thornless shoots of blackberry line PA45m. A False Discovery Rate of 0.05 was used, and the transcripts were filtered for a log2 fold change of ±2.

### RNA-seq expression analysis of anthocyanin biosynthesis structural and regulatory genes

RNA-Seq data for fruit samples of the blackberry cultivar ‘Navaho’ at three developmental stages (green, red, and black) were retrieved from the NCBI Sequence Read Archive (project accession PRJNA744069). Quality control and adapter trimming were performed using Trimmomatic 0.39 [114] to remove low-quality bases. The cleaned reads were pseudoaligned to the coding sequences (CDS) of the BL1 genome using Salmon 1.10.1, with the entire genome used as a decoy to improve alignment accuracy. Gene expression levels were quantified as read counts and normalized using the trimmed mean of M-values (TMM) method in the EdgeR package [118], accounting for variations in library size and RNA composition across samples. The normalized read counts were log-transformed to facilitate visualization and comparison. Anthocyanin structural and regulatory genes were identified by a BLAST 2.15.0 [119] search against the BL1 genome using identified genes in *Arabidopsis* as the query. Pathway genes were further identified by parsing through the genome annotation files. Genes related to the anthocyanin pathway were visualized as heat maps to illustrate their differential expression across the three fruit developmental stages. Key genes in the pathway were identified based on their expression patterns and roles in anthocyanin production. Additionally, transcription factors regulating the anthocyanin biosynthesis pathway were analyzed, with their expression patterns categorized to identify potential activators and repressors, highlighting their regulatory roles during fruit development.

### Analysis of genome resequencing data

Illumina sequences from seven blackberry genotypes (‘Prime-Ark® Freedom’, ‘Prime-Ark® 45’, PA45m, BL2, ‘Prime-Ark® Traveler’, ‘Kiowa’, and ‘Osage’) were trimmed using Trimmomatic/0.39 [114] to remove adapters and low-quality reads. The cleaned reads were aligned to the BL1 assembly with bwa mem (bwa/0.7.17) [120], and SNPs were identified using Freebayes/v1.3.2 [133]. The resulting vcf file was filtered to retain SNPs with a minimum depth of 15 and quality score of 20. All filtered SNPs were input into SnpEff/5.1d [121] to predict the effects of SNPs.

Additionally, the clean reads for all seven genotypes were aligned to the Hillquist genome assembly using the same alignment tool, bwa mem (bwa/0.7.17), followed by SNP calling with Freebayes/v1.3.2. Summaries of the SNP data from the vcf files were generated using the RTGtools package (https://github.com/RealTimeGenomics/rtg-tools).

### Comparative genomics

The predicted proteins from the BL1 blackberry and other related Rosaceae genomes [woodland strawberry: *Fragaria vesca*; diploid blackberry: *Rubus argutus* cv. Hillquist; red raspberry: *Rubus idaeus* cv. Anitra; rose: *Rosa chinensis* ‘Old Blush’; *R*. *chingii*; and black raspberry: *R. occidentalis*) were grouped into orthogroups using OrthoFinder/2.5.2 [122]. A high-confidence phylogenetic tree was generated using the protein sequences of 1847 single-copy orthologs identified with OrthoFinder/2.5.2. Additionally, predicted proteins from each subgenome of the BL1 genome were classified into orthogroups using the same OrthoFinder pipeline.

### Identifying disease resistance and susceptibility genes

Disease resistance genes were identified by scanning the predicted blackberry protein domains with the NBS Hidden Markov Model (HMM) profile using hmmer/3.2.1 [123]. Specific Pfam HMMs were employed to detect NB (PF00931), TIR (PF01582), RPW8 (PF05659), and LRR (PF00560, PF07725, PF13306, PF13855) domains, with domain profiles obtained from the Pfam database (http://pfam.xfam.org/). Pfam hits were filtered using *P* < 1e-04. To identify potential coiled-coil (CC) structures, we used DeepCoil/2.0.1 [124] and set the threshold for CC structure detection at 0.82. For phylogenetic tree construction of NBS-encoding genes, we first aligned the proteins with complete NBS domains using MUSCLE/3.8.31 [125]. We then utilized the alignment for tree construction with RAxM/8.2.10 [134] and visualized the tree using ape [126] and ggtree [127] packages in R. We scanned blackberry protein domains for the MLO HMM profile using hmmer/3.2.1 for domain PF03094. Similarly, DMR6 HMM profile was scanned using the 2OG-Fe(II) oxygenase superfamily (PF03171) domain and the DIOX_N domain (PF14226).

### Transcription factor prediction

Transcripts present within the locus for primocane and thorny were extracted from the BL1 genome. These transcripts were used to identify transcription factors from the Plant Transcription Factor Database (PlantRegMap) [128]. *Arabidopsis* top hit for these transcripts were identified using blastp (−evalue 0.00001) (ncbi_blast/2.10.1) [129] compared with TAIR10 peptides (www.arabidopsis.org).

## Supplementary Material

Web_Material_uhaf052

## Data Availability

The assembly and annotation files of the BL1 blackberry genome have been uploaded to the Rosaceae database (accession number: tfGDR1070). All raw sequencing data are available in the NCBI under BioProject: PRJNA1125996.
